# Relationship between primitive reflexes, functional fitness, handgrip strength, and physical activity in older adults aged 65 and over

**DOI:** 10.14814/phy2.70229

**Published:** 2025-03-28

**Authors:** Erzsébet Stephens‐Sarlós, Anna Horváth‐Pápai, Eliza E. Tóth, Ferenc Ihász, Angéla Somogyi, Attila Szabo

**Affiliations:** ^1^ Faculty of Health and Sport Sciences Széchenyi István University Győr Hungary; ^2^ Doctoral School of Health Sciences, Faculty of Health Sciences University of Pécs Pécs Hungary; ^3^ Doctoral School of Psychology ELTE Eötvös Loránd University Budapest Hungary

**Keywords:** aging, elderly, exercise, fitness, functionality, health

## Abstract

The reemergence of primitive reflexes (PRs) in older adults is associated with dementia and cognitive impairment. Recent experimental work suggests gentle sensorimotor exercises may halt or reverse PR's *inverse development*. These findings question whether physical activity (PA) is negatively related to PRs. This study aimed to test this relationship in 52 older adults aged 66 and over who were volunteers from seven Hungarian nursing homes. They were tested individually using the Senior Test, hand‐grip strength, 13 PRs, and PA levels using the Global Physical Activity Questionnaire. Apart from upper and lower body flexibility, all functional fitness indices and PA were negatively related to the number of PRs. A bootstrapped multiple hierarchical linear regression revealed that only PA was a statistically significant predictor (*p* < 0.001) of the PRs, accounting for 41% of the variance. This study is the first to demonstrate a robust negative relationship between PA and PRs and a weak negative association with hand‐grip strength and four elements of functionality in older adults. The implications of the results could be significant for developing interventions to prevent or delay PRs' inverse development, which is associated with adverse mental health in older adults.

## 1 INTRODUCTION

Primitive reflexes (PRs) are automatic responses regulated by the brainstem. They diminish as cortical control develops after the first six months of life (Zafeiriou, 2004). The PRs are regulated by gamma‐aminobutyric acid (GABA) (Ditmar, 2011; Laliberte et al., 2022; Zafeiriou, 2004), which peak in early adulthood and decline after age 50, potentially causing PRs to reappear in older adults (Porges et al., 2021). Aging also reduces Brain‐Derived Neurotrophic Factor (BDNF), linked to GABA dysregulation and neurodegeneration (Kim et al., 2017). Lower physical activity (PA) with age further decreases BDNF, weakening cortical inhibition and possibly triggering PRs (Sleiman et al., 2016). Their reappearance in older adults is also known as *inverse development* (Altunkalem Seydi et al., 2024) which could signal cortical regulation decline due to brain degeneration, such as ischemic injuries (Boxtel et al., 2006). The presence of PRs is widespread in dementia patients, with a 13.94 to 16.38 times higher prevalence than in healthy individuals (Altunkalem Seydi et al., 2024).

Examples of PRs are the Moro reflex, where a falling sensation triggers arm extension and retraction; the rooting reflex, prompting head‐turning and mouth‐opening when the cheek is touched; and the sucking reflex, triggered by touching the roof of the mouth (Zafeiriou, 2004). The palmar grasp reflex causes fingers to close around an object in the palm, and the plantar grasp reflex triggers toes to curl when the sole is stroked (Zafeiriou, 2004). The asymmetrical tonic neck reflex (ATNR) extends the arm and leg on one side while flexing the opposite side when the head turns, and the symmetrical tonic neck reflex (STNR) fosters crawling by altering limb positioning based on head movement (Zafeiriou, 2004). Other reflexes include the tonic labyrinthine reflex (TLR), which adjusts posture with head tilt; the Galant reflex, curving the body toward one side when the spine is stroked; and the stepping reflex, where stepping motion occurs when held upright with feet touching a surface (Zafeiriou, 2004).

Although it remains uncertain whether PRs naturally reemerge as part of aging, research suggests that their prevalence increases with age, as observed among individuals aged 66–82 in the Maastricht Aging Study (Boxtel et al., 2006). A longitudinal work found that PRs' inverse development was associated with malnutrition risk and aspiration pneumonia in nursing home residents (Hobo et al., 2013). A recent study found that PRs are inversely related to mental health in healthy older adults (Stephens‐Sarlós et al., 2024). Research also shows that PRs are negatively associated with motor functions in children (Gieysztor et al., 2018), but there is no similar research in older adults. Given this population's psychomotor decline and diminished physical functionality (Garber et al., 2010), testing the relationship between PRs and functional fitness in older adults appears to be warranted, especially considering new promising research that sensorimotor exercises might favorably affect PR development (Stephens‐Sarlós et al., 2024).

Physical fitness in older adults is evaluated through strength, flexibility, coordination, and endurance, all essential components of functional fitness—a key indicator of functionality in exercise gerontology (Miotto et al., 1999; Roy et al., 2010). The Fullerton Functional Fitness Test (FFFT), or the Senior Fitness Test (Pepin et al., 2004), assesses functional fitness by measuring upper and lower body strength (FF1), flexibility, coordination, and endurance. Handgrip strength is another proposed index of muscular strength and endurance (Vaidya & Nariya, 2021). Furthermore, evidence suggests that it correlates positively with various elements of functional fitness in older adults (Alonso et al., 2018). Consequently, Migaj et al. (2022) have recently modified the FFFT by adding the handgrip strength assessment.

Therefore, a positive association between PA, handgrip strength, and FFFT scores can be expected (Toth et al., 2024). Additionally, PA may help mitigate the negative impact of PRs (Altunkalem Seydi et al., 2024; Boxtel et al., 2006) on mental health indices by downregulating PR activity (Szabo, 2024). Given these relationships, this study aims to explore the connections between PRs, FFFT, handgrip strength, and PA. Specifically, the research questions are: (1) Which components of the FFFT, if any, are associated with PRs inverse development? (2) Is there a relationship between PRs and handgrip strength in older adults, as this has not been previously studied? Furthermore, (3) Which of these indices can significantly predict PRs' inverse development?

This field study explored the interrelationships between PRs, PA, and functional fitness in older adults to shed light on a potential but unexplored connection between PA, functional living, and PRs as markers of neurodegenerative processes in older adults. By investigating these connections, we seek to pave the road for intervention studies that could modify PRs' inverse development, which are known markers of neurodegenerative dysfunctions in older adults (Altunkalem Seydi et al., 2024; Boxtel et al., 2006). Consequently, the results of this research can potentially inform interventions targeting PR suppression and promote strategies to enhance physical and cognitive health in aging individuals.

## 2 METHODS

### 2.1 Participants and Ethics

We recruited participants from seven nursing homes with the approval of their management. The nursing homes were located in Hungary, specifically in Győr‐Moson‐Sopron and Fejér counties, as well as Budapest's capital. This geographical context is important for assessing the generalizability of the findings. The study, conducted in May 2024, received ethical approval (permission No. DHK‐2024/00039/2, SZE/ETT‐2/2024 (V.6)) from the Research Ethics Board at Széchenyi István University. All (*n* = 52) participants signed an informed consent form, and none of the measures were invasive. There were 16 males and 36 females (mean age = 77.88 ± 6.57), ranging from 66 to 94. The study adhered to the ethical guidelines of the British Psychological Society (2021) and the Helsinki Declaration principles for research with human participants (World Medical Association, 2013). All participants were healthy and received medical clearance for the study, meaning that none of them had an uncontrolled illness, eating or behavioral dysfunction, or viral or bacterial infections. Furthermore, none had a history of pneumonia or malnutrition that could confound the results (Hobo et al., 2013).

### 2.2 Measures

#### 2.2.1 Primitive Reflexes

We assessed PRs using standardized methods outlined by Sanders and Gillig (2011) and Fiorentino (2014) and evaluated 13 specific reflexes, including the STNR, Moro, Galant, ATNR, and TLR. The Babinski reflex was also assessed, as it represents a key PR commonly evaluated in clinical and research contexts to determine neural functionality.

Each reflex was tested in six repetitions on both sides of the body. For example, the Babinski reflex was elicited by drawing a blunt object along the lateral sole in an L‐shaped motion. Similarly, the grasp reflex was tested by applying pressure to the participant's central palm, avoiding interference from the back of the hand. All these reflexes are categorized as PRs, as they reflect the functional integrity of the brainstem and neural pathways (Fiorentino, 2014). Two researchers conducted the testing, with a third present as an observer to maximize consistency and resolve disagreements.

#### 2.2.2 Functional Fitness

The FFFT was based on Jones and Rikli (2000) and Rikli and Jones (1999, 2013). It included six measures:

1. Lower body strength (FF1)—30s chair test, complete stand up and sit down (number of repetitions)
2. Upper body strength (FF2)—lifting 2 (women) or 3.5 (men) kg dumbbell while sitting on a chair and doing full arm bend and stretch (number of repetitions in 30 s)
3. Upper body flexibility (FF3)—fingers touching behind the back (back scratch) (+/− cm)
4. Lower body flexibility (FF4)—forward bend from chair to extended leg (chair sit‐and‐reach) (+/− cm)
5. Complex coordination (agility, balance, and walking speed [FL5])—standing up from a chair and avoiding a buoy 2.44 m away (8 feet) – recording duration in seconds
6. Endurance (FF6)—The six‐minute walk‐in‐place test records the number of total steps completed in 6 min, raising each knee halfway between the patella (kneecap) and iliac crest (top hip bone).

#### 2.2.3 Handrip Strength

Handgrip strength was measured with a CAMRY Model EH101 hand dynamometer manufactured by Zhongshan Camry Electronic Co, Ltd., Zhongshan, China. This instrument is ISO 9001 certified by the Société Générale de Surveillance (SGS) and has a maximum capacity of 90 kg. It automatically captures and displays the peak grip strength achieved during a measurement, enabling consistent tracking of maximum grip power. Due to its high accuracy and reliability, it is widely used in research settings (i.e., Bari et al., 2024). The dynamometer was professionally calibrated based on the manufacturer's specifications to ensure accuracy and consistency.

#### 2.2.4 Physical Activity

We used the Global Physical Activity Questionnaire (GPAQ, V2; World Health Organization, 2021) to assess PA. The GPAQ is a 16‐question tool assessing PA in adults across various domains, including work, transportation, and leisure. It measures the frequency, duration, and intensity of physical activity, helping to classify individuals as sufficiently active or inactive based on MET‐minutes/week. A sample item is: “*In a typical week, on how many days do you do vigorous‐intensity activities as part of your work?*” Its internal reliability (Cronbach's alpha) is between 0.86 and 0.97 (Mead et al., 2008). We recorded the total PA in terms of MET‐minutes/week.

### 2.3 Procedure

Testing occurred individually in the morning in a quiet room in the participants' nursing home. Two researchers conducted the testing in the presence of a third senior researcher who only ensured the correct number of trials in PR and handgrip testing and provided an opinion in case the two experimenters disagreed on a measurement. The tests were performed in a randomized sequence over 4 weeks. While we did not expect order effects in testing, randomization served the practical purpose of testing four individuals in rotating order. Accordingly, FFFT, PR assessment, determination of the handgrip strength, and GPAQ assessment proceeded in ABCD, BCDA, CDAB, and DABC orders, and four researchers carried them out on Mondays, Wednesdays, and Fridays between 8.00 and 12.00 am. Each test was conducted by one researcher, who also entered and verified the data for the specific test in an SPSS file. Subsequently, they were re‐verified for accuracy by two other researchers and then subjected to statistical analyses.

### 2.4 Data analysis

The reported results are based on the raw data openly available at the Mendeley data repository (doi:10.17632/wy3sdt675n.1). We performed two bootstrapped statistical tests (correlation and regression) because we could not reach the calculated minimum (>100) sample size using the G* Power software (Faul et al., 2007). To compensate for this limitation emerging from the difficulty of recruiting independently functioning older adults aged 65 years or over willing to participate in scientific research, we used bootstrapping, a valuable resampling technique for small sample sizes. This method improves estimation accuracy and provides robust confidence intervals without assuming a specific distribution. It also enhances the statistical power and reduces bias by generating multiple resamples from the observed data to better approximate the population distribution (Efron & Tibshirani, 1994).

## 3 RESULTS

The descriptive statistics of the dependent measures are presented in Table 1.

**TABLE 1:** Descriptive statistics of the dependent measures.

	*N*	Mean	Std. deviation
FF1	52	10.77	4.77
FF2	52	15.73	5.53
FF3	52	−7.29	12.75
FF4	51	−5.00	18.82
FF5	52	11.50	6.06
FF6	52	296.19	93.03
PR Right	52	5.25	1.97
PR Left	52	4.44	2.15
Total PR	52	9.69	3.79
Handgrip LR Average	52	20.27	5.91
Physical Activity	52	1013.23	589.07

*Note*: FF1–FF6 are the Fullerton Functional Test six component results.

Abbreviations: Handgrip_LR_Average, The left and right hands averaged handgrip strength; Physical Activity, MET‐minutes/week; PR, primitive reflex.

### 3.1 Correlations

**FIGURE 1:**
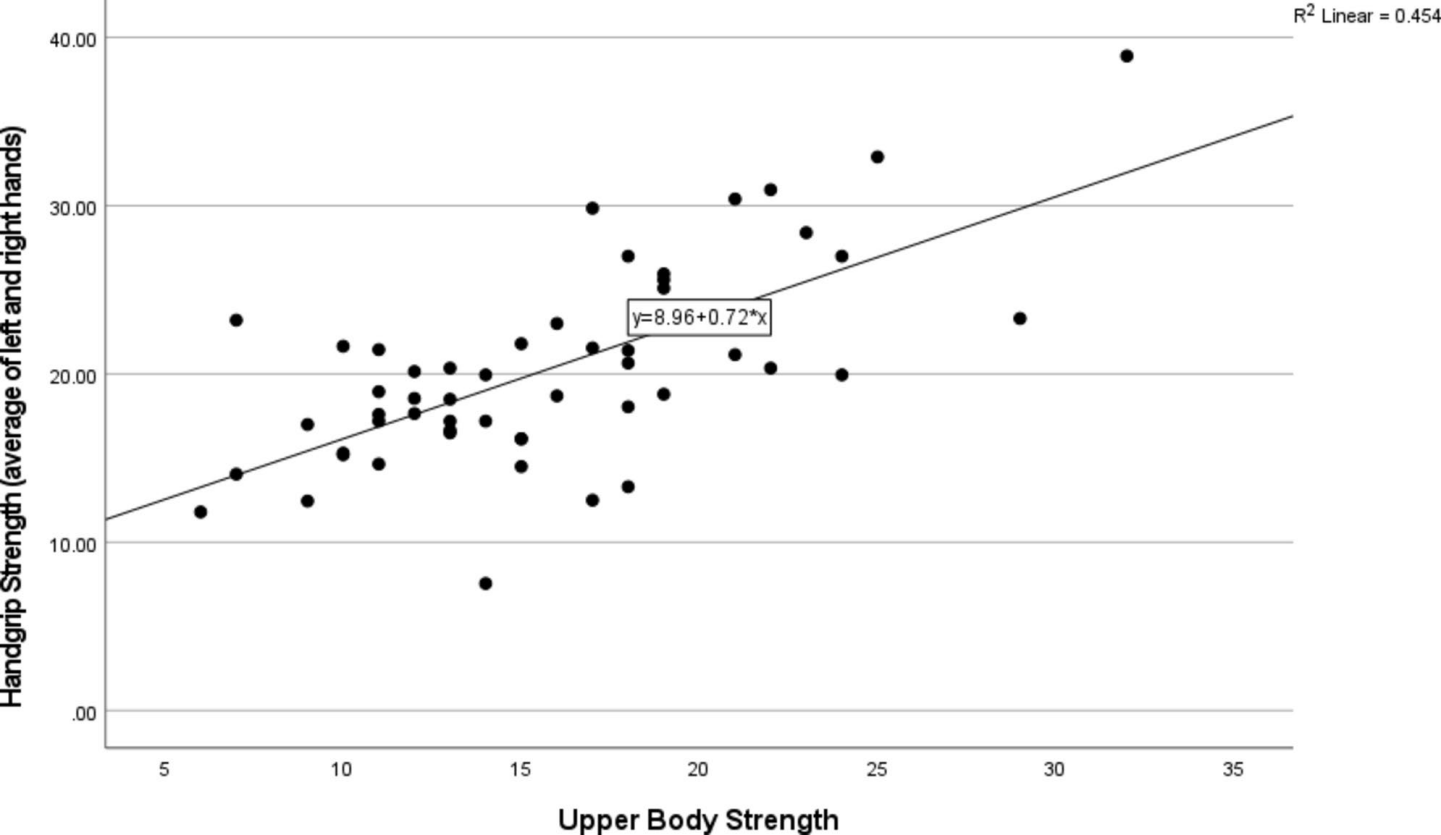
Scatter plot of the relationship between handgrip strength and upper body strength.

To examine the relationships between PRs, FFFT scores, PA, and handgrip force, we calculated bias‐corrected accelerated (BCa) bootstrapped (1000 samples) Pearson's *r* correlations coefficients. The BCa method is robust for estimating confidence intervals because it does not rely on parametric assumptions like normality. It adjusts for bias and skewness in the sampling distribution, making it well‐suited for smaller samples and non‐normal data (Wilcox, 2012). Noteworthy is that handgrip strength correlated statistically significantly (*p* < 0.05) with all measures apart from upper and lower body flexibility (FF3, FF4). The results are presented in Table 2. It should be emphasized that there was a strong positive correlation between handgrip strength and upper body strength (Figure 1).

**TABLE 2:** Correlations (Pearson's *r*) between functional fitness test results, the total number of primitive reflexes, physical activity (MET‐minute/week), and handgrip force.

			FF1	FF2	FF3	FF4	FF5	FF6	PR	PA
F2	*r*		0.615**	‐‐						
	*p*		<0.001							
	Bias^a^		0.005							
	SE		0.127							
	BCa 95% CI	Lower	0.269							
		Upper	0.834							
FF3	*r*		0.218	0.262	‐‐					
	*p*		0.124	0.064						
	Bias		0.008	−0.007						
	SE		0.143	0.144						
	BCa 95% CI	Lower	−0.067	−0.035						
		Upper	0.519	0.520						
FF4	*r*		−0.312*	−0.209	0.109	‐‐				
	*p*		0.026	0.142	0.446					
	Bias		0.036	0.014	0.005					
	SE		0.202	0.166	0.144					
	BCa 95% CI	Lower	−0.657	−0.513	−0.175					
		Upper	0.227	0.190	0.431					
FF5	*r*		−0.787**	−0.480**	−0.282*	0.114	‐‐			
	*p*		<0.001	<0.001	0.045	0.424				
	Bias		−0.005	−0.004	0.003	−0.020				
	SE		0.050	0.131	0.154	0.202				
	BCa 95% CI	Lower	−0.870	−0.685	−0.555	−0.347				
		Upper	−0.705	−0.231	0.034	0.429				
FF6	*r*		0.685**	0.474**	0.326*	−0.083	−0.580**	‐‐		
	*p*		<0.001	<0.001	0.020	0.561	<0.001			
	Bias		−0.002	0.003	−0.007	0.016	−0.004			
	SE		0.060	0.170	0.138	0.179	0.096			
	BCa 95% CI	Lower	0.539	0.068	0.035	−0.418	−0.759			
		Upper	0.789	0.759	0.570	0.314	−0.389			
PR	*r*		−0.397**	−0.321*	−0.039	0.256	0.293*	−0.237	‐‐	
	*p*		0.004	0.022	0.785	0.070	0.037	0.095		
	Bias		−0.004	−0.006	0.001	−0.001	0.000	−0.004		
	SE		0.090	0.133	0.170	0.119	0.109	0.137		
	BCa 95% CI	Lower	−0.548	−0.551	−0.367	0.003	0.047	−0.477		
		Upper	−0.232	−0.067	0.272	0.458	0.498	0.026		
PA	*r*		0.436**	0.248	−0.022	−0.273	−0.341*	0.151	−0.638**	‐‐
	*p*		0.001	0.079	0.881	0.053	0.014	0.290	<0.001	
	Bias		−0.009	0.001	−0.004	0.005	0.006	0.001	0.001	
	SE		0.112	0.125	0.149	0.132	0.102	0.150	0.082	
	BCa 95% CI	Lower	0.187	−0.029	−0.297	−0.506	−0.511	−0.188	−0.772	
		Upper	0.619	0.509	0.252	0.012	−0.127	0.440	−0.485	
HG	*r*		0.403**	0.668**	0.133	−0.118	−0.359**	0.363**	−0.302*	0.314*
	*p*		0.003	<0.001	0.354	0.410	0.010	0.009	0.031	0.025
	Bias		−0.010	−0.010	−0.006	0.013	0.005	−0.005	0.000	0.000
	SE		0.146	0.092	0.149	0.164	0.110	0.174	0.105	0.118
	BCa 95% CI	Lower	0.092	0.475	−0.177	−0.429	−0.556	−0.025	−0.480	0.064
		Upper	0.645	0.810	0.393	0.212	−0.115	0.659	−0.095	0.542

Abbreviations: BCa, bias‐corrected accelerated method; CI, 95% confidence interval; FF1–FF6, Fullerton tests; HG, handgrip strength; PA, physical activity MET‐minute/week; PR, total number of primitive reflexes; SE, standard error.

* = significant 0.05 level (2‐tailed).

** = significant at 0.01 level (2‐tailed).

^a^ Bootstrap bias (1000 samples).

### 3.2 Hierarchical Regression

A BCa bootstrapped multiple hierarchical regression analysis assessed the predictors of PRs. We added variables based on their correlation strength. The Durbin‐Watson statistic (1.504) suggested minimal autocorrelation within acceptable limits. In Model 1, PA significantly predicted PRs, explaining 40.8% of the variance (*R* ^*2*^ = 0.408, *p* < 0.001, *β* = −0.004), with a reliable negative association. When FF1 was added in Model 2, PA remained significant (*p* < 0.001), but the effect size decreased, and FF1 was not a significant predictor (*p* = 0.195). In Model 3, adding FF2 did not improve the model, and neither FF1 nor FF2 were significant. Model 4 showed that adding handgrip strength did not enhance prediction (*p* = 0.966), with PA still significant. In Model 5, FF5 also failed to contribute significantly (*p* = 0.567). Consequently, adding FF1, FF2, handgrip strength, and FF5 to the model did not significantly increase the explained variance beyond PA.

**FIGURE 2:**
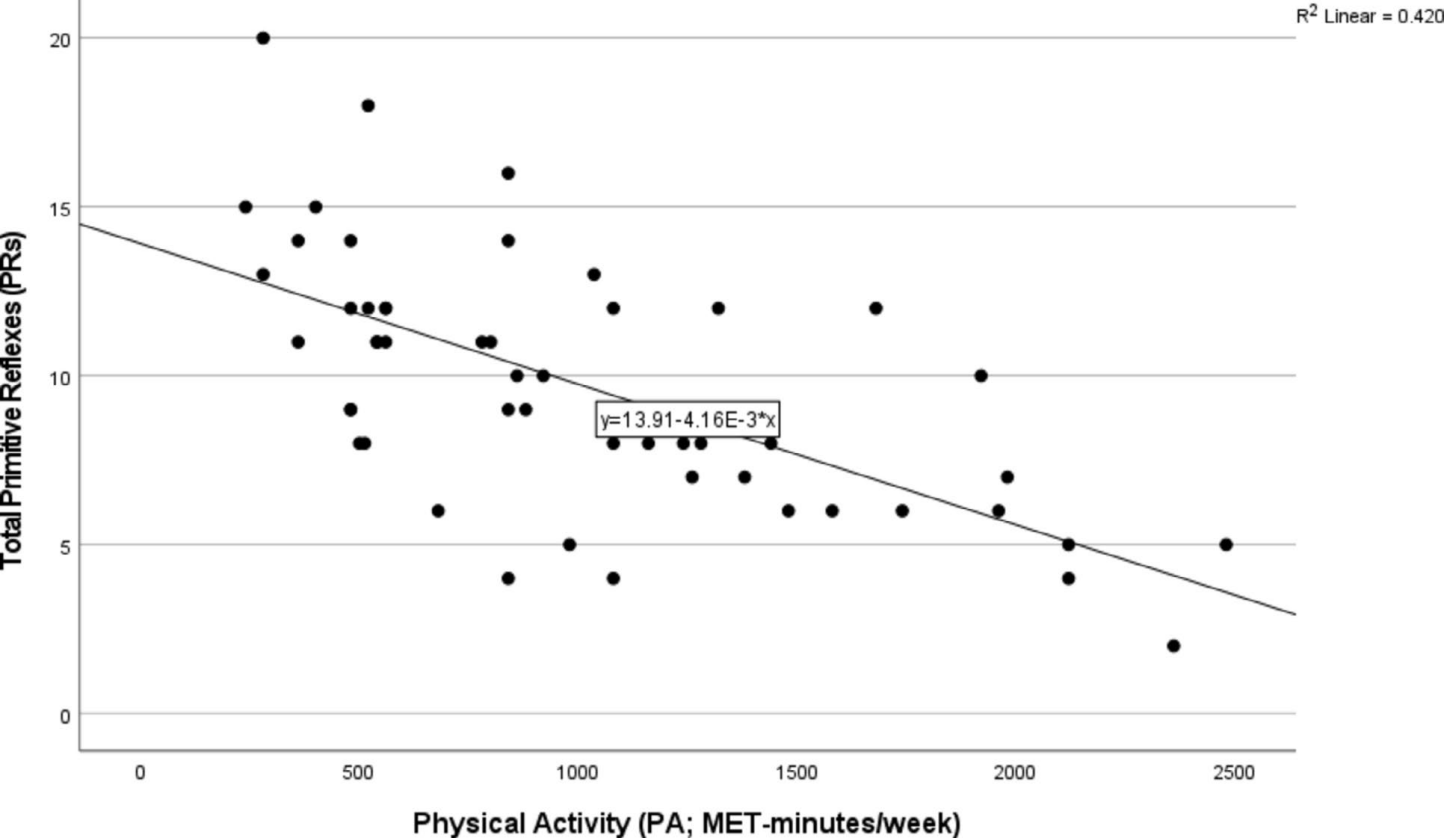
Scatter plot of the relationship between primitive reflexes (PRs) and physical activity (PA) expressed as MET‐minutes/week based on GPAQ.

**FIGURE A1:**
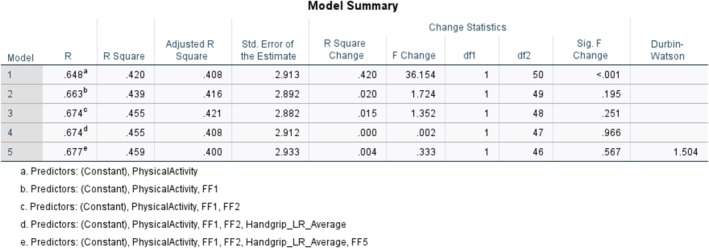
Model summaries.

**FIGURE A2:**
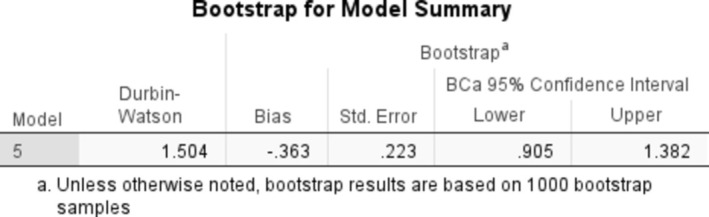
Bootstrap for model summary.

**FIGURE A3:**
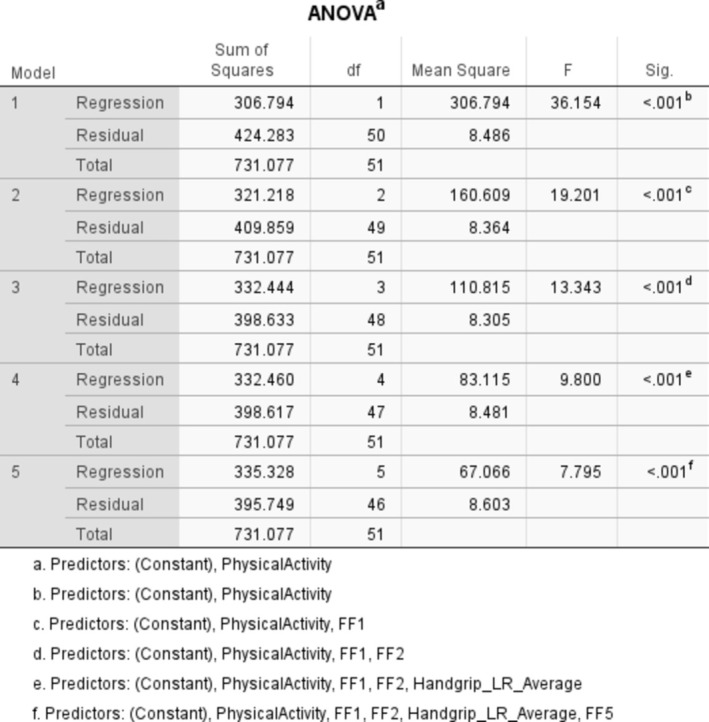
Analysis of variance.

**FIGURE A4:**
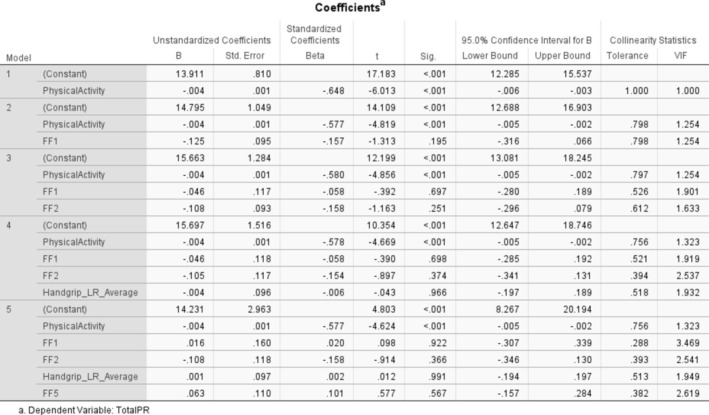
Model coefficients.

**FIGURE A5:**
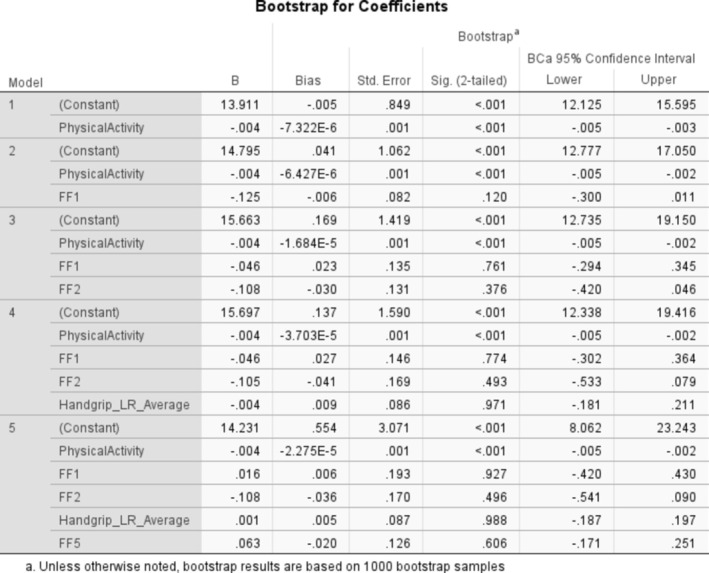
Bootstrap for coefficients.

The bootstrap analysis for Model 1, based on 1000 resamples, confirmed the stability and significance of the regression coefficients. The intercept estimate was 13.911, representing the predicted value of the dependent variable when PA is zero, with a minimal bias of −0.005 and a standard error of 0.849, indicating high precision. The *p*‐value was <0.001, and the 95% bias‐corrected and accelerated (BCa) confidence interval of 12.125 and 15.595 suggested that the true intercept lay within this range. The PA coefficient was −0.004, indicating a negative association with PRs. The bias was negligible (−7.322E‐6), and the standard error was 0.001, showing a precise estimate. The relationship was statistically significant (*p* < 0.001), with the 95% BCa confidence interval ranging from −0.005 to −0.003, confirming a robust negative association. These results indicate that PA is a reliable predictor, with consistent effects across different resampled datasets. The full results of the hierarchical regression are illustrated in Appendix (Figures A1, A2, A3, A4, A5). Figure 2 illustrates the association between PA and PRs.

## 4 DISCUSSION

This is the first study to show that PA level, assessed as MET‐minutes/week, has a strong inverse relationship with the number of PRs in older adults. Indeed, based on Cohen (1988), an *r* correlation coefficient value, which is also an effect size, exceeding 0.50 can be assumed to reflect strong connections. While Toth et al. (2024) showed that a gentle sensorimotor exercise intervention could alter favorable PRs, the connection between PRs and PA levels has not been investigated. Our findings suggest that more active older adults have fewer PRs. Considering that PA levels accounted for about 41% of the variance in PRs, these findings might have substantial implications for preventing neurodegeneration (Altunkalem Seydi et al., 2024; Boxtel et al., 2006) and maintaining positive mental health (Toth et al., 2024) in older adults. While a causal relationship cannot be drawn from the current research, the findings are robust enough to stimulate epidemiologic and other longitudinal investigations in this area.

Although the correlations are statistically significant (refer to Figures 1 and 2), it is important to interpret them within the context of exploratory research. The confidence intervals derived from bootstrapping provide robust estimates, but causal relationships cannot be established without further longitudinal studies. This study's negative correlation between PA and PRs aligns with established evidence that PA elevates GABA and BDNF levels. These biochemical changes enhance cortical inhibition, which is critical for mitigating the reemergence of PRs in older adults (Kim et al., 2017; Sleiman et al., 2016).

Furthermore, knowing the connection between PRs and GABA (Ditmar, 2011; Laliberte et al., 2022; Zafeiriou, 2004), PA and BDNF (Sleiman et al., 2016), and that PA increases GABA (Maddock et al., 2016) as well as BDNF (Walsh et al., 2020), the current results should promote future basic research in this area. Indeed, the mechanism of the here‐observed inverse relationship between PRs and PA merits scholastic attention through neurobiochemical and neuroimaging research to understand the underlying processes. The results of such works could contribute to applied knowledge in preventing or delaying physical and mental degeneration in older adults.

While PA and PRs were also related to lower body strength (FF1) and complex coordination (FF5), such results could be expected based on PA levels. For example, higher PA could be associated with stronger upper body strength and better complex coordination while being inversely related to PRs. Still, despite significant correlations, the effect sizes (*r*) were small to medium, which explains why these measures were not significant predictors of the PRs in the examined regression model.

Handgrip strength correlated with all except upper and lower body flexibility measures (see Table 2), confirming that it is a good index of physical functionality in older adults (Kim et al., 2022). However, its strongest correlation emerged with upper body strength, which predicted about 45% of the variance later (refer to Figure 1). In line with our results, previous studies have found a strong positive association between handgrip strength and muscle strength in older adults, indicating that this simple and inexpensive measure can serve as a reliable index for assessing overall physical capacity, health, and even the risk of death (Duchowny et al., 2017; Koopman et al., 2014; Roberts et al., 2013).

In this study, handgrip strength also correlated inversely (see Table 2) with the number of PRs, but the strength of the relationship was only moderate. Since the number of PRs in older adults reflects cognitive degeneration (Altunkalem Seydi et al., 2024), their negative correlation with handgrip strength might suggest that the latter could also be associated with cognitive functions. This surmise is supported by a scoping review (Fritz et al., 2017) reporting that handgrip strength is a global indicator of muscle strength, and a lower handgrip strength is associated with reduced cognitive performance over time, while a higher handgrip strength is protective against cognitive decline. These authors suggest that monitoring handgrip strength in people with cognitive impairment may be helpful in disease progression and prognosis.

Our findings suggest that promoting PA in older adults may be pivotal in mitigating PRs' inverse development. By increasing GABA and BDNF levels, PA might enhance cortical inhibition, thereby counteracting the neural mechanisms underlying PR reemergence. While supported by existing biochemical evidence, this relationship warrants further investigation to establish causal pathways.

This study highlights the importance of integrating PA into cognitive and motor health interventions in older adults. However, specific recommendations, such as optimal intensity or duration of PA, cannot yet be derived from this dataset. Future longitudinal and interventional studies should explore these parameters to provide actionable guidelines.

### 4.1 Limitations

The study has limitations that must be considered when interpreting the results. First, the results are based on independently functioning Hungarian volunteer adults aged 65 and over, who may not represent this population. Despite objective measures of PRs and FFFT, PA was estimated based on subjective reports. Furthermore, due to the low number of male participants, gender differences could not be tested in this research. Finally, causal connections cannot be drawn from the current study.

## 5 CONCLUSIONS

This is the first study to show a robust inverse relationship between PRs and PA. The results also revealed a moderate correlation between PRs and handgrip strength. Still, only PA emerged as a strong predictor of PRs, accounting for 40.8% of the variance in the latter. While the mechanism(s) of the relationship remains to be determined in future empirical research, the current results are promising for recommending PA as a possible preventive measure against PRs' inverse development and, hence, cognitive decline in older adults.

## FUNDING INFORMATION

The authors did not receive financial support for this research.

## CONFLICT OF INTEREST STATEMENT

The authors declare no conflicts of interest.

## ETHICS STATEMENT

The research was conducted with ethical approval (permission No. DHK‐2024/00039/2, SZE/ET‐T‐2/2024 (v.6)).

## CONSENT

All participants signed an informed consent form, and none of the sampling was invasive.

## USE OF AI

Apart from grammar checks, no AI was used to write this paper.

## APPENDIX A

The results of the bootstrapped multiple hierarchical regression (extract of the results output from the SPSS analysis) (Figures A1, A2, A3, A4, A5).
